# Impact of Different Transportation Modes on the Transmission of COVID-19: Correlation and Strategies from a Case Study in Wuhan, China

**DOI:** 10.3390/ijerph192315705

**Published:** 2022-11-25

**Authors:** Danwen Bao, Liping Yin, Shijia Tian, Jialin Lv, Yanjun Wang, Jian Wang, Chaohao Liao

**Affiliations:** 1 College of Civil Aviation, Nanjing University of Aeronautics and Astronautics, Nanjing 211106, China; 2 School of Transportation, Southeast University, Nanjing 211189, China; 3 Air Traffic Management Bureau of Central South of China, Guangzhou 510422, China

**Keywords:** transportation modes, transmission pattern, quantile regression, negative binomial regression, control efficiency

## Abstract

Transportation is the main carrier of population movement, so it is significant to clarify how different transportation modes influence epidemic transmission. This paper verified the relationship between different levels of facilities and epidemic transmission by use of the K-means clustering method and the Mann–Whitney U test. Next, quantile regression and negative binomial regression were adopted to evaluate the relationship between transportation modes and transmission patterns. Finally, this paper proposed a control efficiency indicator to assess the differentiated strategies. The results indicated that the epidemic appeared 2–3 days earlier in cities with strong hubs, and the diagnoses were nearly fourfold than in other cities. In addition, air and road transportation were strongly associated with transmission speed, while railway and road transportation were more correlated with severity. A prevention strategy that considered transportation facility levels resulted in a reduction of the diagnoses of about 6%, for the same cost. The results of different strategies may provide valuable insights for cities to develop more efficient control measures and an orderly restoration of public transportation during the steady phase of the epidemic.

## 1. Introduction

Coronavirus disease 2019 (COVID-19) was first detected in Wuhan, Hubei Province of China, at the end of 2019. The human-to-human transmission nature of the virus was officially announced by the government on 20 January 2020. Three days later, the central government of China imposed a lockdown in Wuhan to quarantine the center of the epidemic. Public transportation services were severely restricted in Wuhan at that time, including the closure of train stations, airports, and major highways. However, an estimated 5 million people were reported to have left Wuhan before the implementation of the lockdown, to celebrate the Lunar New Year, resulting in rapid spread of the virus throughout the country [1]. As one of the major transportation hubs in China, Wuhan is well connected to many other cities [2]. It has been universally demonstrated that population movement is a major factor that influences the spread of this kind of infectious disease, with outflows from Wuhan playing a key role [3].

Previous studies have evaluated the relationship between the spread of COVID-19 and air transportation [4,5,6], railway transportation [7,8,9] and road transportation [1,10]. Nevertheless, the existing studies have not clarified the mechanism of how different transportation modes affect epidemic transmission. Therefore, it is crucial to understand how different transportation modes contribute to epidemic transmission, and, thus, more reasonable traffic restriction strategies can be proposed.

The main contributions of this paper are reflected in the following aspects: (1) to reveal the correlation between levels of transportation facilities and epidemic transmission, we employed K-means clustering with Mann–Whitney U testing; (2) before clarifying the degree of impact, we first quantified the role of different transportation modes in the transmission, by two indicators (i.e., transmission speed and pandemic severity), and then fit them by quantile regression and negative binomial regression, respectively; (3) we examined the implementation effect of prevention strategies considering transportation modes and facilities levels in order to provide a scientific foundation for urban epidemic prevention.

The remainder of the paper is organized as follows: Section 2 discusses the related literature. Section 3 provides the collected data and describes the modelling approach. Section 4 reports the results. Section 5 proposes the related prevention strategies. Section 6 presents the discussion. Conclusions are drawn in Section 7.

## 2. Literature Review

The world has been hit by several infectious diseases this century, including SARS, H1N1, MERS and COVID-19. Many academics are interested in how transportation affects the transmission of infectious diseases. Air transportation was found to accelerate the transmission of SARS significantly [4]. Additionally, accessibility to the airline network substantially impacted the spread speed of SARS [4,6,11]. Chang et al. [11] verified the relationship between H1N1 and air transportation. The H1N1 virus is more contagious in locations near national roads and highways than it is in other parts of the country [12]. The severity of impact of the H1N1 virus also varies by transportation modes at different stages of transmission [10]. Since COVID-19’s epidemiological traits differ greatly from those of infectious diseases such as SARS and H1N1, some researchers have conducted further studies. Liu et al. [13] proposed that the COVID-19 pandemic posed significant challenges to public transport operators. Thus, some scholars conducted targeted studies for different transportation modes. Zhao et al. [7] discovered a significant correlation between railway transportation and epidemic spread. A study by Wan et al. [9] confirmed that railway systems accelerated epidemic transmission. In addition, Chen et al. [8] constructed a cities’ network in Northeast China and employed the spreading dynamics model to simulate the spread of infectious diseases based on the data of Baidu Migration. Oztig et al. [14], Liu et al. [5], and Colizza et al. [15] studied the role of air transportation in epidemic transmission. They discovered a strong connection between flight volume and the number of confirmed cases. In addition, curtailing flights was a crucial component of containing epidemic spread. Further, Zhu et al. [16] discovered that railway transportation had a greater impact than air transportation. Road transportation was considered in research by Zhang et al. [1] and Lu et al. [17]. They pointed out that air and railway transportation had a particularly significant impact on epidemic spread compared to road transportation. Additionally, the “counterfactual” concept was also employed by some researchers to examine the relationship between transportation modes and epidemic spread [18,19]. The above studies have demonstrated the differential impact of various transportation modes on epidemic spread. However, the extent of this effect has not been quantified, especially under multiple transportation modes.

Researchers have looked further into the link between urban transportation facilities and epidemic spread. Browne et al. [20] concluded from overall findings that airports are highly susceptible to epidemic. By simulating the spread of epidemic through transportation hubs, Xu et al. [21] illustrated the significance of these hubs in epidemic transmission. According to Gaskin et al. [22], areas closer to transit stops had higher case numbers and fatality rates. Kutela et al. [23] sought to explore the long-term impact of COVID-19 on transportation facilities. They found that responses instituted for economic recovery and public health were less likely to be long-term, while responses meant to improve safety or bicycle/pedestrian mobility were more likely to be long-term. Most of the previous studies defined transportation facilities as the presence of transportation stations within the city. However, neither the transportation volume nor the function of transportation facilities has been considered. Thus, it is not possible to develop differentiated prevention strategies according to the level of transportation facilities. This paper attempts to address the issues by identifying key transportation hub cities that influence epidemic transmission, as well as evaluating the implementation effect under diverse prevention strategies.

This paper describes the epidemic spread pattern and creates regression models from two perspectives: speed and severity. Due to discrepancies in the evaluation dimensions of the above two indicators, different regression methods needed to be selected. Standard ordinary least squares (OLS) regression only reflects the conditional mean, as well as requiring stricter distribution of the dependent variable. Due to significant variability in the transmission speed of the epidemic disease in different cities, OLS regression models fit poorly [17]. Another commonly used model is the proportional hazards model (COX). It is applied to continuous variables such as survival status and survival time, but is a poor fit for discontinuous variables such as transmission speed [22]. Quantile regression estimates the independent variable based on the conditional quantile of the dependent variable. It can achieve an accurate fit to the full range of dependent variables [24]. Therefore, this paper adopted quantile regression to quantify the influence of different transportation modes on the transmission speed. The regression for transmission severity is a counting regression problem. The typical counting regression model is the Poisson regression model, but due to issues such as data complexity, it is challenging to obtain precise findings in practical situations [14]. Hence, we utilized a generalized model of the Poisson regression model, i.e., negative binomial regression model, to overcome these problems. Table 1 demonstrates the synthesizing papers on research topics.

## 3. Materials and Methods

### 3.1. Research Region

**Figure 1:**
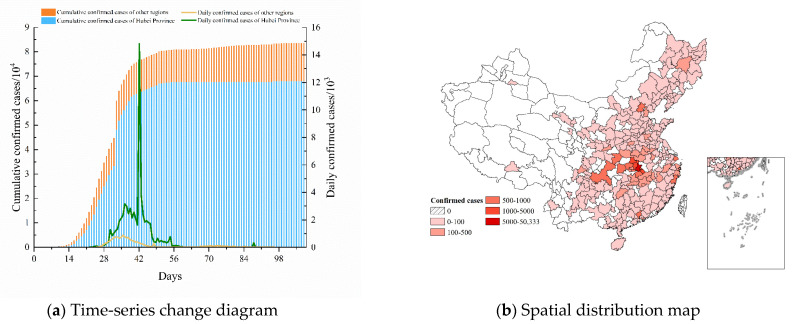
Spatial and temporal distribution of COVID-19 in China: (**a**) time-series change diagram; (**b**) spatial distribution map.

This paper focuses on the volume of air, railway, and road transportation from Wuhan to other cities during the initial phase of the epidemic outbreak. After excluding cities with no confirmed cases in the studied duration or with missing data, 310 cities were eventually selected as the research region. The daily confirmed cases and the cumulative confirmed cases were collected from 11 January to 28 April 2020. Figure 1 shows the temporal and spatial distribution of COVID-19 in China.

Since the emergence of COVID-19 in Wuhan, confirmed cases have emerged in various cities in China. The cumulative number of confirmed cases displays an “S-curve” growth pattern (Figure 1a). According to Ai et al. [2], more than 5 million people left Wuhan between 10 January 2020 and the commencement of the lockdown. By 31 January, the disease had spread to approximately 300 cities, following an east-to-west evolutionary trend (Figure 1b). One of the main factors contributing to the epidemic spread was the outflow from Wuhan. Thus, tracking people from Wuhan was an essential task for each city, to contain the transmission. This paper mainly focused on the epidemic data from 11 January to 29 February 2020 due to the fact that more than 90% of the cases at that time had a history of travel to the epicenter [1]. Although community transmission had started to occur between 20 February and 29 February, the overall percentage was relatively small. This paper employed robustness testing to prevent the impact of this small-scale data on the fluctuation of the results.

### 3.2. Data Sources

In this paper, three transportation modes were mainly considered, i.e., air, railway and road. The flight data were from the OAG database. The railway data were from the China Train Time Query website (https://qq.ip138.com/train, accessed on 8 May 2022). The road data were from the China Bus Schedule Query (https://www.keyunzhan.com/qicheshikebiao, accessed on 8 May 2022). The daily confirmed cases and the cumulative confirmed cases were obtained from the National Health Commission of China and the provincial health commissions.

### 3.3. Definition of Epidemic Transmission Patterns Indicators

To reflect the epidemic transmission pattern more accurately, two indicators are defined in this paper for measurement of the transmission pattern.

(1) Transmission speed: the number of days between the announcement of the first confirmed case of COVID-19 in Wuhan (11 January 2020) and the first confirmed case in that city (Equation (1));
(2) Transmission severity: the cumulative number of confirmed cases in a given city (until the end of February 2020) (Equation (2)).

(1) $$v_{i}=t_{i}-t_{0}$$

(2) $$s_{i}=\overset{T}{\underset{1}{\sum }}m_{1}+m_{2}+\cdots +m_{T}$$

where $v_{i}$ and $s_{i}$ denote the transmission speed and severity in city *i*, respectively; $t_{0}$ and $t_{i}$ denote the time of the first confirmed case in Wuhan and the time when the first confirmed case appeared in city *i*, respectively; and $m_{T}$ is the number of confirmed cases in city *i* on day *T.*

### 3.4. Classification of Transportation Facilities and Inspection Methods

(1) K-means clustering algorithm

The simplicity and effectiveness of the K-means clustering method outweigh its drawback, which is the need for pre-determined K values. The fundamental principle of K-means clustering is to calculate the distance between each data point and each cluster center after selecting initial K cluster centers at random, and then placing each data point in the closest cluster center [25].

The specific process of the algorithm is as follows:

Step 1: Initialize a partition randomly or based on some prior knowledge. Calculate the cluster prototype matrix $M=\left[\begin{array}{ccc} m_{1} & \ldots & m_{k} \end{array}\right]$.

Step 2: Assign each object in the data set to the nearest cluster.

Step 3: Recalculate the cluster prototype matrix based on the current partition.

Step 4: Repeat steps 2–3 until there is no change for each cluster.

The process will continue until the termination condition is satisfied. The distortion function can be used to determine the change in cost resulting from each iteration (Equation (3)).

(3) $$J\left(x_{1},x_{2},\cdots x_{m};\mu_{1},\mu_{2},\cdots \mu_{K}\right)=\frac{1}{m}\overset{m}{\underset{i=1}{\sum }}{\left(x_{i}-\mu_{c\left(i\right)}\right)}^{2}$$

where x represents data points, μ represents the cluster centres, and $\mu_{c\left(i\right)}$ represents the nearest cluster centroid.

Analogously to the algorithm in this paper, we counted and categorized the number of transportation facilities (i.e., airports, railway stations, and bus stations) in 310 cities in terms of transportation volume and socio-economic characteristics (i.e., population, GDP).

We first gave the convergence for different K values under the same convergence condition and found that the algorithm was more likely to reach convergence at a K value of 2. Table 2 shows the iterative results. Meanwhile, according to previous studies on the impact of different transportation facilities on epidemic transmission [1,10,26], the K value was set to 2 generally. Thus, we set the K value to 2 comprehensively. The K-means clustering algorithm was implemented in SPSS version 23.0.

As can be seen from the table, convergence was achieved when the clustering centers did not change after 7 iterations. This demonstrated that convergence could be reached faster for a value of K of 2. Therefore, this paper divided different transportation facilities into two categories.

(2) Mann–Whitney U test

To assess the effect of the level of transportation facilities on epidemic transmission, further testing of the data from both groups was required. Conventional parametric tests such as t-test are mainly applied to single-group data tests. Moreover, the data distribution must satisfy normality and variance chi-square [27]. Therefore, we verified the normality of the data using the Kolmogorov–Smirnov test. The statistical results indicated that the data did not follow a normal distribution because they were significant at the 1% level. Thus, the Mann–Whitney U test with 95% confidence level was chosen to investigate the correlation between the level of transportation facilities and epidemic transmission.

The procedure for this test is as follows.

Step 1: Mix the two sets of data and arrange the ranks in order of size. The smallest data rank is 1, and so on.

Step 2: Find the rank sum of the two samples separately $W_{1}$,$\;W_{2}$.

Step 3: Calculate the Mann–Whitney U test statistic for the two samples (i.e., $n_{1}$,$\;n_{2}$), respectively (Equations (4) and (5)).

(4) $$U_{1}=n_{1}n_{2}+\frac{n_{1}\left(n_{1}+1\right)}{2}-W_{1}$$

(5) $$U_{2}=n_{1}n_{2}+\frac{n_{2}\left(n_{2}+1\right)}{2}-W_{2}$$

Choose the smaller of $U_{1}$ and $U_{2}$, then compare with the critical value $U_{A}$. When $U<U_{A}$, reject $H_{0}$ and accept $H_{1}$.

Step 4: Make a judgement.

### 3.5. Modelling the Impact of Transportation Modes on the Epidemic Transmission

#### 3.5.1. Epidemic Transmission Patterns

(1) Transmission speed

**Figure 2:**
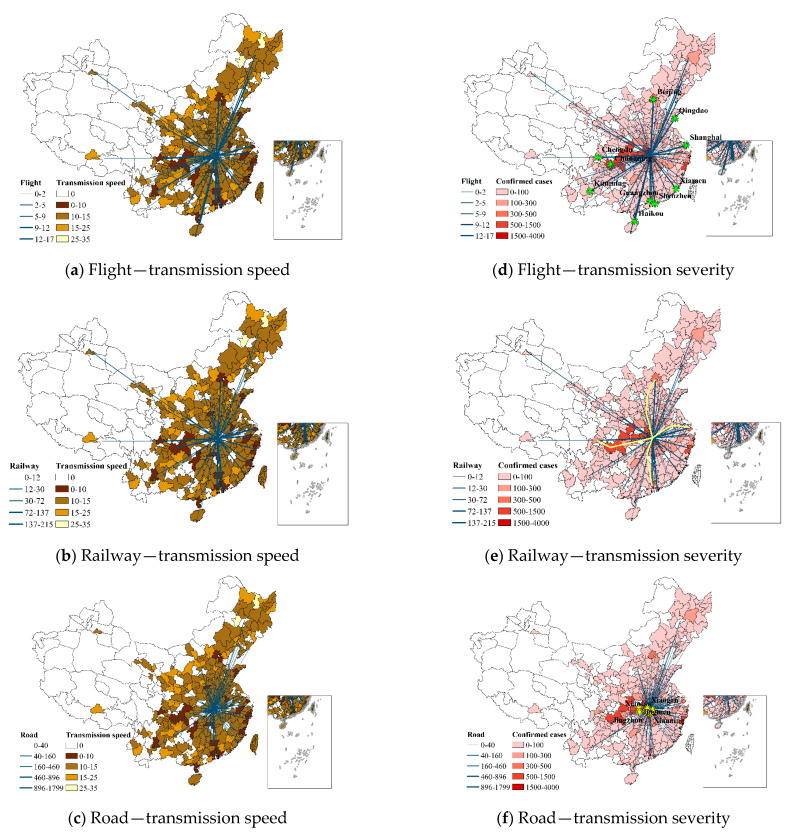
Correlation between different transportation modes and transmission patterns.

Figure 2 illustrates how closely the amount of transportation leaving Wuhan is related to the transmission speed of the epidemic. Overall, the epidemic occurred early in cities near Wuhan, mostly because road transportation is crucial to epidemic transmission (Figure 2c). Roads such as the Wu-Huang Expressway and the Han-Cai Expressway enabled the epidemic to spread across cities in Hubei Province earlier. Around 15% of the total road transportation volume is comprised of road transportation between Wuhan and Huanggang. However, cities far away from Wuhan (e.g., Shenzhen, Beijing, Shanghai, Chengdu, etc.) experienced the epidemic within a short period of time (approximately 7–9 days) due to air and railway transportation (Figure 2a,b). Due to Wuhan’s share of air and railway transportation volume being up to 25% and 30%, respectively, with such cities, the epidemic quickly expanded to those locations.

(2) Transmission severity

As can be seen in Figure 2, the more cumulative diagnoses there were, the more tightly Wuhan was connected to that city by transportation. The cities with frequent air transportation are in the Beijing–Tianjin–Hebei region, the Pearl River Delta region, etc. This demonstrates that the epidemic is more likely to spread to megacities via air transportation (Figure 2d). Wuhan is situated at a significant node of China’s high-speed railway system (Figure 2e), which provides fast access to many cities. This is also in line with the epidemic spread pattern identified by Cui et al. [28]. Cities with more road transportation are mostly concentrated in Hubei Province, which indicates that road transportation plays an important role in short-distance transmission.

#### 3.5.2. Regression Modelling of the Transmission Speed and Severity

(1) Quantile regression

Previous studies have shown the variability in the timing of the occurrence of the first confirmed cases in different cities. Therefore, it was necessary to study the extent to which different transportation modes affected the transmission speed. Common conditional mean regression is more demanding on the data set itself, as it requires the dependent variable to adhere to conditions such as normal distribution. Additionally, it is prone to interference from outliers. Quantile regression is an extension of traditional linear regression, which can accurately fit the complete distribution of the dependent variable [29], and this model is more robust to outliers [24]. Therefore, we utilized a quantile regression model to clarify the influence of different transportation modes on the epidemic transmission.

Separate fits were performed for the interquartile point range of 0.05–0.95 to carefully measure the variation in impact (0.1 per interval). Sajadi et al. [30] showed that COVID-19 virus is more likely to spread in cities between 30° N and 50° N. Accordingly, city latitude and longitude coordinates were also included as covariates. The final model variables were determined as shown in Table 3. Considering that the orders of magnitude of population and GDP were much larger than other variables, we referred to the method in the existing studies [1,14] to obtain their logarithms to avoid their error on the model fit.

We developed a multivariate quantile regression model to estimate the association between the quantile of the epidemic transmission speed and different transportation modes in each city (Equation (6)).

(6) $$\begin{array}{ll} Q_{t}\left(y_{i}\right)=\beta_{0}\left(t\right)+ & \beta_{1}\left(t\right)\times lnx_{1}+\beta_{2}\left(t\right)\times lnx_{2}+\beta_{3}\left(t\right)\times x_{3}+\beta_{4}\left(t\right)\times x_{4}\\ & +\beta_{5}\left(t\right)\times x_{5}+\beta_{6}\left(t\right)\times x_{6}+\beta_{7}\left(t\right)\times x_{7}+\beta_{8}\left(t\right)\times x_{8}\\ & +\beta_{9}\left(t\right)\times x_{9}+\beta_{10}\left(t\right)\times x_{10} \end{array}$$

where $y_{i}$ denotes the time of first confirmed cases in different cities (i=1,2,⋯310); t denotes the different quantile points; $\beta_{0}\left(t\right)$ denotes the intercept at different quantile points; and $\beta_{2}\left(t\right),\;\cdots \;\beta_{10}\left(t\right)$ is the regression coefficient at different quantile points.

(2) Negative binomial regression

We were also interested in the effect of each variable on the transmission severity. However, the essence of the problem was to build a count-based regression model. The basic count regression model is the Poisson regression model. Practical issues can be challenging to match with the count regression model due to problems such as data complexity [31]. To build the model, the variance of the data must equal the mean value, which is often challenging to meet in real situations. Furthermore, the regression line deviates more from reality when the ratio between the variance and the mean is high, resulting in excessive observational heterogeneity [32].

To analyze the variation in the cumulative number of confirmed cases across cities, we adopted the negative binomial regression model. This model is based on the initial Poisson distribution by introducing the Gamma distribution. We selected this method since the variance of dependent variable was greater than the mean (Table 3). Thus, it was applicable to solve this problem.

To ascertain whether the model was applicable, we first performed an over-discrete O test on the data. The test results are displayed in Table 4.

The findings of the test evidently showed that the data were too discrete because the absolute value of O value was more than 1.96 (*p*-value less than 0.05). Thus, the negative binomial regression model was adopted to overcome the problem of discrete data. The modelling process is explained as follows.

(7) $$P\left(Y_{i}=y_{i}\right)=\frac{\mu_{i}^{y_{i}}exp\left(-\mu_{i}\right)}{y_{i}!}$$

where P(·) demonstrates the probability of Y confirmed cases observed in city i over a specified period (until 29 February); $y_{i}$ can take the values 0,1,2,⋯; and $\mu_{i}$ denotes the expected infected frequency for city i.

As a function of the variables in the regression model, the $\mu_{i}$ parameter is estimated as follows.

(8) $$ln\left(\mu_{i}\right)=x_{i}^{T}\beta$$

where β is a vector of estimated coefficients of exploratory variables including the mentioned variables in Table 3. A vector of coefficients is then estimated by maximizing the likelihood function.

(9) $$lnL\left(\beta \right)=\underset{i}{\sum }\left[-exp\left(x_{i}^{T}\beta \right)+\left(x_{i}^{T}\beta \right)y_{i}-lny_{i}!\right]$$

The least squares estimation for the negative binomial regression model has a significant error rate [32]. Therefore, the Gauss–Newton iterative algorithm was used for the maximum likelihood estimation. The core idea of the Gauss–Newton iterative method is to use the Taylor series expansion to approximate the nonlinear regression model instead. This allows the regression coefficients to approximate the best nonlinear regression coefficients.

The least squares method is used to directly solve the system of equations in order to obtain the optimal solution. However, it is frequently challenging to put into practice in real settings. In contrast, the more popular Gauss–Newton method iteratively approaches the ideal answer step by step. In addition, the least squares method is generally only applicable to linear regression.

A high variance-to-mean ratio is frequently caused by heterogeneity among observations, resulting in overdispersion. The Poisson regression assumption is relaxed by including an additional randomness term in Equation (8) which is gamma-distributed.

(10) $$ln\left(\mu_{i}\right)=x_{i}^{T}\beta +\varepsilon_{i}$$

where $\varepsilon_{i}$ follows gamma distribution with mean $\mu_{i}$ and variance α. The NBR distribution has a mean $\mu_{i}$ and variance $\mu_{i}+\alpha \mu_{i}{}^{2}$, where α is the overdispersion parameter. The model for analyzing the variation of the dependent variable is proposed as follows.

(11) $$\begin{array}{ll} ln\left(s\right)=\beta_{0}+\beta_{1} & \times \left(lnx_{1}\right)+\beta_{2}\times \left(lnx_{2}\right)+\beta_{3}\times x_{3}+\beta_{4}\times x_{4}+\beta_{5}\times x_{5}\\ & +\beta_{6}\times x_{6}+\beta_{7}\times x_{7}+\beta_{8}\times x_{8}+\beta_{9}\times x_{9}+\beta_{10}\times x_{10} \end{array}$$

where $\beta_{0}$ represents the intercept of the negative binomial regression model; and $\beta_{1},\beta_{2},\cdots \beta_{10}$ represents the regression coefficient of the corresponding variable.

## 4. Results

### 4.1. Division of Transportation Hubs

**Figure 3:**
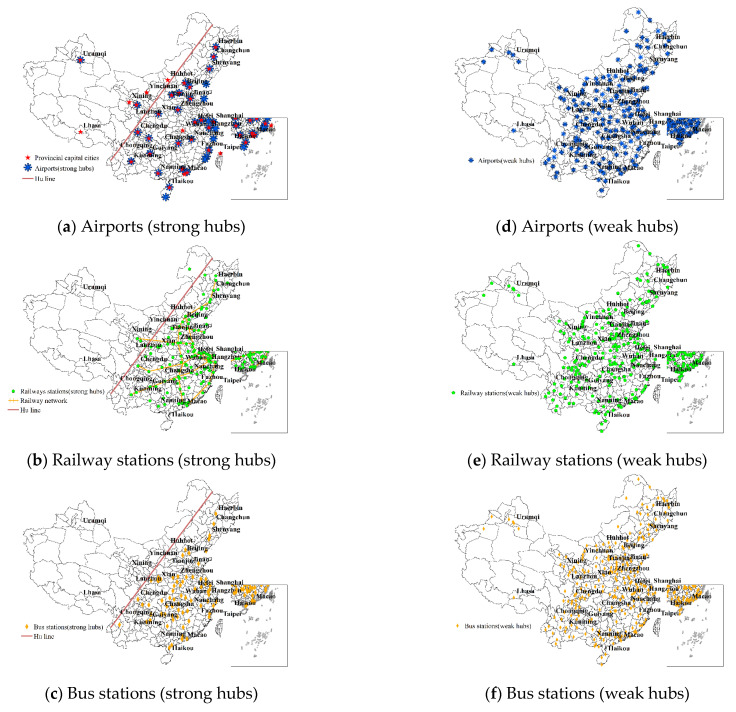
Distribution of different levels of transportation facilities.

Figure 3 displays the clustering and classified results for airports, railway stations, and bus stations using the division method in Section 3.5.

The strong hubs were located mainly in the eastern region and roughly bordered by the Hu Huanyong line (i.e., a comparison line proposed by geographer Hu Huanyong to classify the population density of the country).The weak hubs were scattered in small and medium-sized cities in various provinces. Combined with the spatial distribution of the epidemic (Figure 2), it can be found that cities with strong hubs reported the first case earlier, and had a higher cumulative number of confirmed cases.

Compared to the strong airport hubs, strong hubs of railway and bus stations were much more densely distributed. Nearly 36% of cities have train stations which were classified as strong hubs, while the number for bus stations and airports were 23% and 11%, respectively. Mostly in megacities with a population of 10 million or more, airports that were classified as strong hubs are shown in Figure 3a with a dotted distribution pattern. This allowed the epidemic to spread from Wuhan to distant provinces via air transportation. The railway stations that were classified as strong hubs demonstrated dispersion belt distribution, as well as extending along the national high-speed railway network of four vertical and four horizontal (Figure 3b). This feature allowed the epidemic to spread to the eastern region. Figure 3c presents the aggregate cluster distribution at the strong hubs of bus stations, which was the primary factor in epidemic transmission to cities near Wuhan.

### 4.2. The Role of Transportation Hubs in the Epidemic Transmission

**Figure 4:**
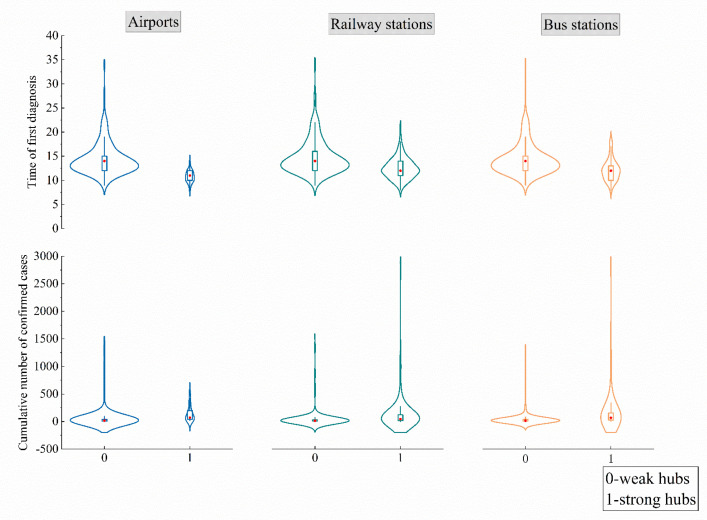
Distribution of the epidemic transmission in two levels of transportation facilities.

The distribution of the time of first confirmed cases, and the cumulative number of confirmed cases in cities with strong and weak transportation hubs, are shown in Figure 4. The curves in the figure from left to right depict the aforementioned information for airports, railway stations and bus stations, respectively.

As can be seen in Figure 4, the curves of both sets of plots exhibited a single-peaked distribution. Cities with strong hubs reported confirmed cases earlier and in greater numbers. Given that the distribution densities of railway and bus stations between cities were comparable (Figure 3), both kinds of facilities had similar effects on epidemic transmission. Table 5 shows the results of the Mann–Whitney U test.

According to the test results, the strong hubs of all three transportation facilities generally accelerated the epidemic’s emergence. The degree to which strong hubs of various transportation facilities influenced the epidemic transmission, varied. Cities with strong airport hubs experienced initial cases 3 days earlier than those without them (median: 11 days/14 days; *p* < 0.01). Similarly, cities with strong railway and bus station hubs experienced the emergence of the epidemic 2 days earlier (median: 12 days/14 days; *p* < 0.01).

Similarly, the presence of strong hubs could aggravate epidemic transmission. The cumulative number of confirmed cases in cities with strong hubs of airports, railway stations and bus stations were 4.2 times (median: 71/17; *p <* 0.01), 3.1 times (median: 44/14; *p <* 0.01) and 4.4 times (median: 66/15; *p* < 0.01), respectively, higher than in other cities.

### 4.3. Influence of Transportation Modes on Epidemic Transmission

(1) Transmission speed

To analyze the factors influencing the time of the first confirmed case, we calculated the quantile regression coefficients (Table 6). The magnitude and significance of the regression coefficients differed between the quantiles, and these quantiles corresponded to 10, 12, 14, and 17 days, respectively.

The three transportation modes had different effects on the epidemic transmission speed. At the lower and middle quantiles (0.25 and 0.55), air and road transportation were substantially and adversely correlated with the epidemic spread speed. Additionally, the coefficients of air transportation were generally more significant than the coefficients of railway transportation. This indicated that the higher the volume of air and road transportation, the earlier the emergence of the epidemic in the cities, where the epidemic emerged in about two weeks. The time of the first case appeared 0.1–0.2 days earlier for every 1% increase in air transportation volume. However, the coefficients of railway transportation were not significant. Compared to railway transportation, road transportation was the primary factor of epidemic transmission in China.

There was no correlation between the number of airports and bus stations and the transmission speed of the virus. Moreover, the number of railway stations was significant at the 0.05 quantile (10 days), suggesting that cities with more than two railway stations were likely to experience the epidemic earlier.

GDP and latitude coordinates were significantly correlated with the time of the first case at all quantiles, mostly at the 0.01 level. GDP was significantly and negatively correlated with transmission speed, while the latitude coordinate was strongly and positively linked with transmission speed. In addition, the longitude coordinate demonstrated a significant negative correlation at the 0.25, 0.55, and 0.85 quantiles, probably due to Wuhan’s closer ties with the eastern region.

**Figure 5:**
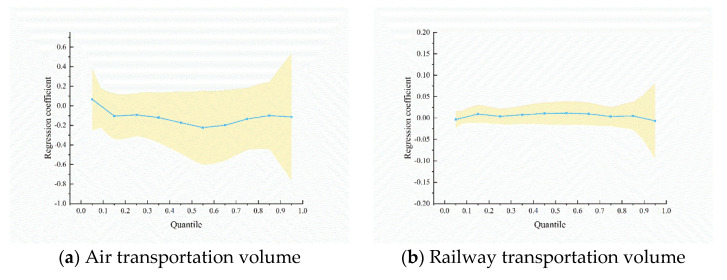

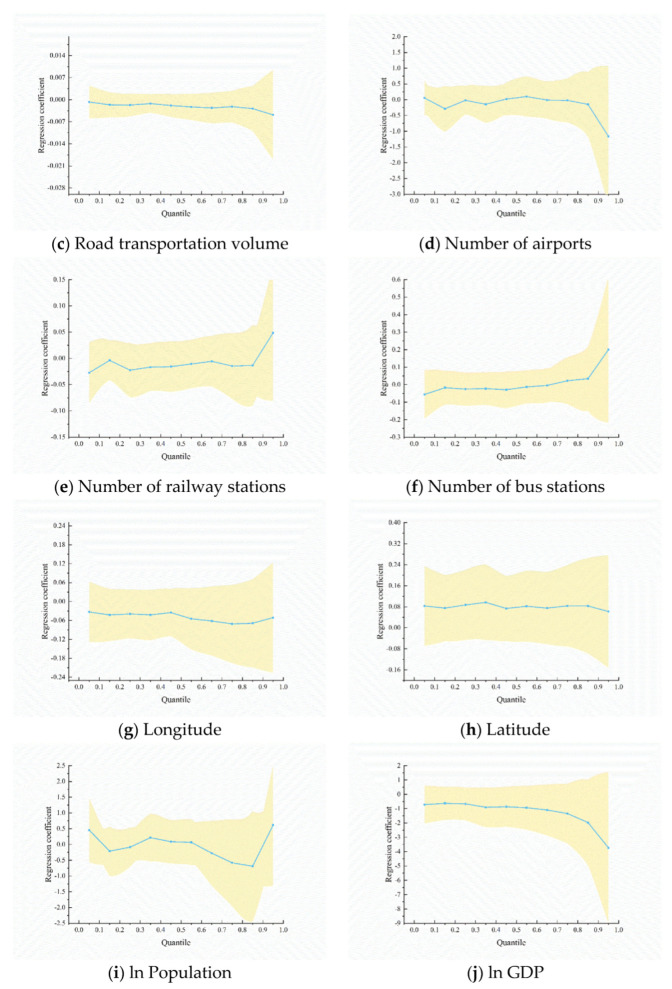
The regression coefficients for quantiles of variables.

The regression coefficients of different variables for the quantiles are depicted in Figure 5. The solid line in the graph shows the change in the quantile coefficient, and the shaded area is the 95% confidence interval. The figure illustrates that the coefficient of air transportation volume declined first, then gradually climbed after the 0.5 quantile, while the coefficient of road transportation volume gradually decreased. As the number of days increased, the negative impact of road transportation on the transmission speed increased, whereas the influence of air transportation reduced. The coefficients of longitude and latitude remained stable between the quantiles. Additionally, the GDP coefficient demonstrated a large decline at the high quantile (0.7), suggesting that as the number of days increased, the transmission speed of the virus in developed cities became faster.

(2) Transmission severity

Table 7 displays the negative binomial regression results for the cumulative number of confirmed cases on January 31 and February 29, respectively, with the regression results on January 31 serving as the robustness test.

The transmission severity of the epidemic was strongly correlated with railway and road transportation. The cumulative number of confirmed cases rose by 0.011% and 0.0065% for every 1% increase in coach and railway travel, respectively. Although road transportation played a major role in the epidemic transmission, railway transportation was more relevant to transmission severity. Air transportation was not related to transmission severity. Due to the low number of domestic flights from Wuhan, the number of cases infected by air transportation in other cities was small. The total number of confirmed cases was not significantly impacted by the number of airports, railway stations and bus stations, which was in line with the factors affecting the transmission speed.

The relationship between the cumulative number of confirmed cases and GDP was positive, suggesting that the epidemic spread more quickly in provincial capital cities or prosperous cities. Both the latitude and longitude of cities showed significant correlations. It has been confirmed that the epidemic is more likely to spread in cities with high temperatures [30]. There was no significant correlation between urban population and the cumulative number of confirmed cases.

## 5. Evaluation of Different Prevention Strategies

According to the previous analysis, it was the transportation volume that influenced whether the epidemic would spread, rather than the presence of airports, railway stations, or bus stations in a city. Additionally, the impact of different transportation modes on epidemic transmission was variable. The prevention strategies of China have proven that cutting off the transportation connections to cities with infections promptly and completely, is the most efficient way to contain the epidemic. During the severe outbreak, such measures were undoubtedly necessary. However, cutting off the transportation links of the city during a stable period would be economically disastrous. Developing a more precise and differentiated prevention strategy can help address this issue. Therefore, we propose the following prevention strategies: a strategy that takes the differences in transportation modes into account (Strategy A), and another strategy considering the level of transportation facilities based on Strategy A (Strategy B). We propose control efficiency indicators to compare the effects of the complete blocking strategy (Strategy C) with the prevention outcomes of the above strategies.

(1) Strategy A

**Figure 6:**
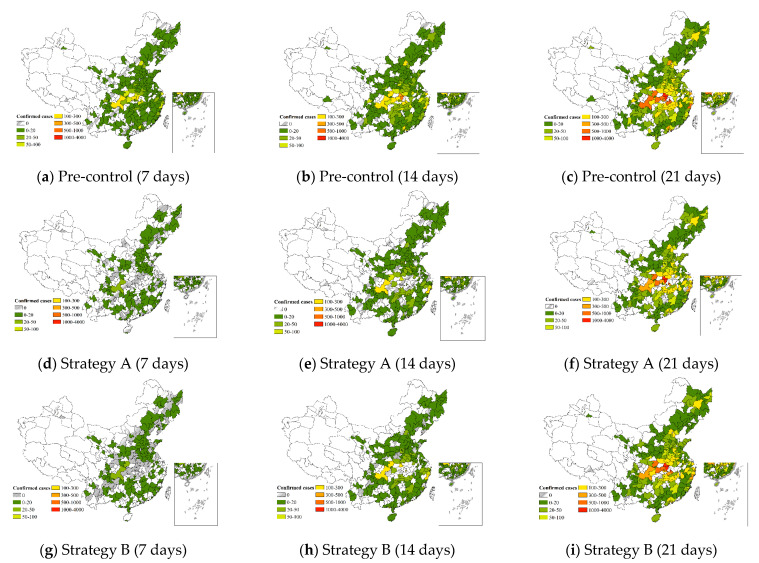
Changes in the epidemic under different strategies.

The preceding study showed a significant correlation between railway and road transportation and the cumulative number of confirmed cases, while the relationship was not close for air transportation. We suggest keeping air transportation as essential, but canceling the necessary railway and road transportation. In Strategy A, equal amounts of railway and road transportation are canceled, and the level of cancellation is classified as medium (50% trips) or high (80% trips). Air transportation is cut in half. Figure 6 displays the fitted epidemic transmission at various time intervals (7 days, 14 days, and 21 days). Table 8 lists the number of infected cities, as well as the number of confirmed cases for various intervals of infection under different prevention levels.

Taking the high prevention level as an example, the change in the number of infected cities showed that the low infection level (0–100) decreased most significantly, proving that cutting off the connection with infected cities could contain the epidemic more quickly for these cities. There was an overall decrease of approximately 36% in the number of confirmed cases across the 310 cities. As can be seen from Table 6, the proportional decrease in the middle and high infection levels was nearly 20% higher than in the low infection levels, indicating that the control effect was more pronounced in the more severe cities. This was particularly significant for cities within Hubei Province. For example, Jingzhou showed a drop of more than 80% in the number of confirmed cases when a suspension of road transportation was adopted. The number of confirmed cases also decreased by roughly one-fifth in Wenzhou and Chongqing, the cities outside Hubei Province with the worst extraterritorial infections.

(2) Strategy B

According to the study mentioned above, cities with strong hubs were significantly associated with the epidemic spread. Therefore, we considered the level of transportation facilities, based on Strategy A. In Strategy B, we adopted Strategy A’s cancellation ratio for cities with strong hubs, while cities without strong hubs were reduced to half. Figure 6 shows the fitted epidemic transmission at various time intervals (7 days, 14 days, and 21 days). Similarly, Table 9 demonstrates the number of infected cities, as well as the number of confirmed cases for various intervals of infection under different prevention levels.

Taking the high prevention level as an example, the number of infected cities in the low infection level (0–100) declined the most. A large base of infected people makes it difficult to clear the epidemic quickly in cities with middle and high infection levels. Over the 310 cities, there was an overall decrease in confirmed cases of approximately 28%. Table 9 shows that the infection level that had a greater decrease under this strategy was the medium infection level (300–500). The cities under the medium infection level were Beijing, Shanghai, Shenzhen, and other megacities with multiple types of hubs. Thus, the control effect of the strategy considering transportation facilities was more apparent.

(3) Comparison of control effects

We proposed control efficiency indicators in combination with the intensity of transportation connections and the number of confirmed cases [17], as shown in Equation (12). Among the indicators was the cost of containing the epidemic in terms of a decrease in the intensity of transportation links; a lower value of this indicator denoted a more expensive and ineffective means of controlling the epidemic.

(12) $$E=\frac{\left(s_{b}-s_{a}\right)/s_{a}}{T_{a}+T_{t}+T_{r}}$$

where $s_{b}$, $s_{a}$ denotes the cumulative number of confirmed cases before and after control, respectively; and $T_{a}$, $T_{t}$ and $T_{r}$ denote the proportion of control for the three transportation modes, respectively.

The calculated results for the three strategies are shown in Table 10. The percentage drop in the total number of confirmed cases was largest for Strategy C, smallest for Strategy B, and median for Strategy A. This proves that completely cutting off transportation links is the most effective way to contain epidemic spread, but is also achieved at the greatest cost. According to the calculations for control efficiency, Strategy B had the highest control efficiency while Strategy C had the lowest. This illustrates that the prevention strategy that takes the level of transportation facilities into account is the least costly. Strategy B used the least amount of transportation control to reduce the cumulative number of confirmed cases by the same percentage. In comparison to Strategies A and C, the percentage of transportation control elimination was 6% and 8%, respectively. In this way, transportation links among some of the cities with weak hubs can remain.

## 6. Discussion

To explore the roles of different transportation modes in the transmission of COVID-19, this paper used transmission speed and severity to characterize COVID-19 and focused-assessed the impact of transportation-related elements on it.

### 6.1. Impact of Transportation Hubs on the Epidemic Transmission

According to the above results, there was variability in the influence of different levels of transportation hubs on the epidemic transmission.

- Transmission speed

Generally, the presence of a strong airport hub had a more pronounced impact on the epidemic transmission speed. Most cities with airports are megacities, which are more conducive to the movement of people. Although the distribution density of airports was substantially lower than the others (Figure 3), their presence may have accelerated the emergence of the epidemic.

- Transmission severity

Among the three types of transportation facilities, strong bus station hubs had a greater influence than the other two categories of facilities. Combined with the transmission characteristics of road transportation, the number of trips undertaken from strong bus station hubs had greater influence, thus, accelerating the epidemic transmission.

### 6.2. The Role of Transportation Modes in the Epidemic Transmission

The quantile regression results indicated that air and road transportation were strongly linked with the transmission speed. This confirmed that the epidemic was primarily transmitted by airports to cities with medium and long distances from the epicenter. Most people returning from Wuhan during the Spring Festival chose long-distance bus travel due to the low cost and high frequency of bus services. Therefore, the main transportation mode that caused the epidemic to spread from Wuhan to Zhejiang, Guangdong, and other provinces, was by road transportation.

Additionally, we found that the number of airports and bus stations did not correlate with the transmission speed. This was mostly due to the wide disparity in the level of urban development in China. Despite the fact that many midwestern cities have developed transportation hubs such as bus stations and airports, the actual size of population mobility is small because of the sparse population and inadequate transportation capacity. This was the fundamental motivation that inspired this paper to categorize transportation facilities according to transportation volume, population, and GDP.

However, the number of railway stations did correlate with the transmission speed. This was mainly due to the fact that most of the cities located in the central-eastern part of China have two railway stations. The first cases in these cities generally emerged earlier.

In addition to transportation-related factors, we found that GDP was negatively correlated with transmission speed. This suggests that the first case will appear earlier in developed cities. The latitude coordinate was positively linked with the transmission speed, implying that initial cases will occur earlier in warmer cities. The longitude coordinate exhibited a significant negative correlation at most quantile points. According to Ni [33], approximately 170,000 people from Wenzhou work in Wuhan. On the eve of the Spring Festival, a sizable number of people returned to Wenzhou, causing the epidemic to spread quickly.

The negative binomial regression results showed that railway and road transportation were closely related to transmission severity. Despite the fact that air transportation could exacerbate the epidemic transmission, railway transportation remained the major factor affecting the transmission severity. During the Spring Festival, the railway schedule is intensive, and the passenger volume is higher than by air transportation.

Additionally, the population did not demonstrate a strong correlation with transmission severity. Therefore, a larger population does not necessarily mean more infections. The most crucial factor was dependent on the transportation connections between Wuhan and that city.

### 6.3. Suggestions Regarding Prevention Strategies

This paper proposes two prevention strategies: the first takes into account the differences in transportation modes (Strategy A) and the second considers the levels of transportation facilities based on Strategy A (Strategy B).

Comparing the two strategies with the complete blocking strategy, we discovered that epidemic transmission can be promptly cut off by a complete blockade in the early phase of the epidemic. By focusing on regulating air and road transportation, the epidemic transmission speed can be slowed down. Differentiated prevention strategy can be established according to the level of transportation facilities when the epidemic reaches a stable period. In key cities, road and railway transportation should be controlled first, followed by air transportation. Since railway transportation has a weaker influence on the epidemic spread than road transportation, relaxing restrictions can be given priority once the epidemic is under control.

## 7. Conclusions

In this paper, we examined the role of the level of transportation facilities in epidemic transmission. Although cities with strong hubs tended to experience the first infection earlier, there was no significant correlation between the number of airports and bus stations, and epidemic spread. People in cities with more than two railway stations were more likely to be infected, thus, prevention efforts should be concentrated there.

The role of three transportation modes, i.e., air, railway, and road, on the transmission speed and severity of the epidemic, was also studied. Railway and road transportation were strongly related to the cumulative number of confirmed cases, while air and road transportation were significantly associated with the time of the first confirmed case. Additionally, latitude and longitude, as well as GDP, had a more substantial impact on the epidemic transmission pattern.

Two types of prevention strategies were proposed and evaluated, which considered the level of transportation facilities and the differences in various transportation modes. Approximately 5% difference was found between the two types of prevention strategies. When the epidemic enters a lull, implementation of the second kind of strategy can lessen the adverse effects of prevention efforts on other facets such as the economy and city transportation.

Our study has several limitations. Firstly, it is mainly related to the scale and quality of the dataset. There may exist improvement in the model accuracy, as more precise data cannot be made public by relevant authorities. Secondly, we investigated the mechanisms of the epidemic transmission via different transportation modes during the external imported phase. We still need to explore, in depth, the mechanism by which the epidemic spreads within the city after its initial arrival, in order to develop an intra-city prevention strategy. In addition, it is also essential to integrate with external control measures of the city to minimize the impact of the epidemic on residents’ lives. Finally, future research should improve the model by taking into account the features of various people and the infectious disease dynamics model.

## Figures and Tables

**Table 1:** Comprehensive papers on research topics.

Categories	Subjects	Epidemic	Findings	References
Influence of transportation modes on epidemic transmission	Air transportation	SARS	(a) Airline network accessibility was a vital variable in determining the spread of SARS; (b) The larger the throughput of the airport city in the Chinese aviation network, the stronger the spreading ability of the node as the source of infection.	[4,6]
		H1N1	The results confirmed that there was a definite relationship between H1N1 and air travel. Additionally, the prevention measures were effective in containing the spread of virus.	[11]
		COVID-19	(a) The findings suggested a positive relationship between higher volume of airline passenger traffic carried in a country and higher numbers of patients with COVID-19; (b) The study provided concrete evidence that the severe curtailing of flights had a spontaneous impact in controlling the spread of COVID-19; (c) The air transportation networkproperties were responsible for the global pattern of emerging diseases.	[5,13,14]
	Railway transportation	COVID-19	(a) The results indicated that the prevalence of COVID-19 infection was 40% higher in cities connected to Wuhan with HSR than in the rest of the cities; (b) The epidemic mainly spread along the axis of “Three Horizontal and One Vertical” with population flow.	[8,9]
	Air, railway and road transportation	H1N1	(a) Proximity to airports and being intersected by national highways or freeways, but not railways, were variables associated with the presence of the disease in a county; (b) Both air and road travel played a significant role in accelerating the spread during phases I and II, but rail travel was only significant during phase II.	[10,12]
		COVID-19	(a) There was strong and significant association between travel by train and the number of cases; (b) The findings demonstrated that high-speed rail and air connectivity with Wuhan resulted in 25.4% and 21.2% increases in the average number of daily new confirmed cases, respectively; (c) The outcomes suggested that frequencies of air flights and high-speed train services out of Wuhan were significantly associated with the number of COVID-19 cases in the destination cities; (d) The results indicated that the most effective way to prevent the coronavirus from spreading quickly and extensively was to control the routes linked to the epicenter at the beginning of the pandemic.	[1,7,15,16]
The role of transportation facilities in epidemic transmission	Airports	SARS	Airports were highly susceptible to the epidemic, from overall findings.	[20]
	Transit stops	COVID-19	Areas closer to transit stops had higher case numbers and fatality rates.	[22]
	All transportation facilities		The findings showed that responses instituted for economic recovery and public health were less likely to be long-term.	[23]

**Table 2:** Iterative results.

Iteration	Change of Clustering Center								
	K = 2		K = 3			K = 4			
	1	2	1	2	3	1	2	3	4
1	282.861	472.872	197.474	190.283	223.367	126.837	44.238	72.258	157.254
2	16.424	119.873	2.135	25.949	87.703	11.968	94.562	3.557	43.450
3	9.339	58.619	5.356	22.958	22.871	0.907	47.560	3.904	44.634
4	1.674	11.558	6.477	28.990	44.285	0.000	37.307	7.528	23.667
5	4.910	32.456	6.246	21.258	19.936	0.907	26.226	6.446	22.286
6	3.243	19.649	3.064	7.354	0.000	0.876	26.038	6.098	19.781
7	0.000	0.000	3.008	12.162	20.092	0.881	33.784	15.498	0.000
8	/	/	2.974	11.754	18.702	1.739	9.610	7.484	0.000
9	/	/	1.971	4.409	0.000	1.690	5.149	4.876	0.000
10	/	/	1.965	4.312	0.000	2.520	0.000	4.035	0.000

**Table 3:** Variable properties.

Variable	Sample Size	Symbols	Average Value	Standard Deviation	Minimum Value	Maximum Value
Population	310	$x_{1}$	15.06	0.73	12.51	17.35
GDP	310	$x_{2}$	25.95	0.96	23.03	28.97
Air transportation volume	310	$x_{3}$	0.95	2.70	0	17
Railway transportation volume	310	$x_{4}$	7.78	27.08	0	215
Road transportation volume	310	$x_{5}$	37.48	178.34	0	1799
Number of airports	310	$x_{6}$	0.65	0.67	0	4
Number of railway stations	310	$x_{7}$	9.38	10.52	0	83
Number of bus stations	310	$x_{8}$	7.59	5.51	1	52
Longitude	310	$x_{9}$	113.17	7.97	80.27	131.16
Latitude	310	$x_{10}$	32.80	6.82	18.25	52.34
Time of first diagnosis	310	v	13.89	3.55	8	34
Cumulative number of confirmed cases	310	s	97.41	316.70	1	3427

**Table 4:** Overdispersion *O* Test.

Sample Size	Average	Variance	*O*-Value	*p*-Value
310	97.4065	100,295.7954	12,786.0753	0.000

**Table 5:** Results of the Mann–Whitney U test.

Indicators	Type	Classification	Median	25% Quantile	75% Quantile	U-Value	z-Value	*p*-Value
*v*	Airport	1	11	10	12	1032.5	−7.6324	0.000 ***
		0	14	12	15			
	Railway station	1	12	11	14	6445	−6.1305	0.000 ***
		0	14	12	16			
	Bus station	1	12	10	13	4918.5	−5.4228	0.000 ***
		0	14	12	15			
*s*	Airport	1	71	39	197	1673	−6.2875	0.000 ***
		0	17	7	42			
	Railway station	1	44	17	120	5906	−6.7931	0.000 ***
		0	14	6	34			
	Bus station	1	66	26	155	4067	−6.6629	0.000 ***
		0	15	7	38			

Note: *** *p* < 0.01.

**Table 6:** Results of quantile regression.

Variables	0.05 (10 Days)	0.25 (12 Days)	0.55 (14 Days)	0.85 (17 Days)
Population	0.4547	−0.0849	0.0688	−0.6891
GDP	−0.7139 **	−0.6634 ***	−0.936 ***	−1.9686 ***
Air transportation volume	0.0663	−0.0924 **	−0.2222 ***	−0.0996
Railway transportation volume	−0.0034	0.0039	0.0115	0.0051
Road transportation volume	−0.0007	−0.0017 *	−0.0023 **	−0.0028
Number of airports	0.0566	−0.0192	0.1001	−0.1466
Number of railway stations	−0.0273 *	−0.0224	−0.0106	−0.0132
Number of bus stations	−0.0558	−0.0253	−0.0126	0.034
Longitude	−0.033	−0.0393 **	−0.0547 **	−0.0688 *
Latitude	0.0833 **	0.087 ***	0.0821 ***	0.0833 *
R^2^	0.1297	0.1558	0.1774	0.2390

Note: * *p* < 0.1, ** *p* < 0.05, *** *p* < 0.01.

**Table 7:** Results of negative binomial regression.

Variables	$s_{220}$		$s_{131}$	
	$\alpha_{i}$	OR Value	$\alpha_{i}$	OR Value
Population	0.2064 (1.6001)	1.2293	0.1935 (1.4179)	1.2135
GDP	0.4684 *** (4.0841)	1.5975	0.5187 *** (4.3070)	1.6798
Air transportation volume	0.0210 (0.6920)	1.0212	0.0194 (0.6303)	1.0196
Railway transportation volume	0.0105 *** (3.3774)	1.0106	0.0113 *** (3.5985)	1.0113
Road transportation volume	0.0065 *** (17.8622)	1.0065	0.0050 *** (13.5560)	1.005
Number of airports	0.0643 (0.6083)	1.0664	0.0311 (0.2793)	1.0316
Number of railway stations	−0.0085 (−1.2351)	0.9915	−0.0081 (−1.1043)	0.9919
Number of bus stations	−0.0111 (−0.7008)	0.9889	−0.0085 (−0.5214)	0.9915
Longitude	0.0331 *** (3.8528)	1.0337	0.0254 *** (2.7176)	1.0257
Latitude	−0.0275 *** (2.6051)	0.9729	−0.0465 *** (4.1733)	0.9546

Note: *** *p* < 0.01.

**Table 8:** Control effects of Strategy A.

Infection Level	Prevention Level	Number of Infected Cities before Control	Number of Infected Cities after Control	Cumulative Number of Cases before Control	Cumulative Number of Cases after Control	Decline Percentage
0–100	High	265	245	6287	4959	21.12%
	Medium		254		5337	15.11%
100–300	High	26	26	4276	3113	27.20%
	Medium		26		3549	17.00%
300–500	High	5	5	1977	1119	43.40%
	Medium		5		1440	27.16%
500–1000	High	8	7	5950	3132	47.36%
	Medium		8		4164	30.02%
1000–4000	High	6	6	11706	6947	40.65%
	Medium		6		8732	25.41%

**Table 9:** Control effects of Strategy B.

Infection Level	Prevention Level	Number of Infected Cities before Control	Number of Infected Cities after Control	Cumulative Number of Cases before Control	Cumulative Number of Cases after Control	Decline Percentage
0–100	High	265	253	6287	5248	16.53%
	Medium		256		5474	12.93%
100–300	High	26	26	4276	3317	22.43%
	Medium		26		3607	15.65%
300–500	High	5	5	1977	1248	36.87%
	Medium		5		1519	23.17%
500–1000	High	8	8	5950	4295	27.82%
	Medium		8		4761	19.98%
1000–4000	High	6	6	11706	7181	38.66%
	Medium		6		8878	24.16%

**Table 10:** Comparison of control effects.

Prevention Strategies	Prevention Level	Cumulative Number of Cases before Control	Cumulative Number of Cases after Control	Decline Percentage	Control Efficiency
A	High	30,196	19,271	36.18%	0.181
	Medium		23,223	23.09%	0.184
B	High		21,288	29.50%	0.236
	Medium		24,239	19.73%	0.235
C	High		19,156	36.56%	0.152

## Data Availability

The flight data are from the OAG database. The railway data are from the China Train Time Query website (https://qq.ip138.com/train, accessed on 8 May 2022). The road data are from the China Bus Schedule Query (https://www.keyunzhan.com/qicheshikebiao, accessed on 8 May 2022).
